# Wry Neck Cured without Cutting

**Published:** 1853-03

**Authors:** Gurdon Buck

**Affiliations:** Surgeon to New York Hospital; 121 Tenth-st


From the New York Medical Times.

*Wry Neck Cured Without Cutting.*

THE success obtained in the following cases of Distortion of the Head, commonly known as Wry Neck, induces the undersigned to make them known to the profession, in order that the treatment employed, which it is believed has not hitherto been applied in such cases, may be fully subjected to the test of experience:

CASE FIRST.

Hester Higgins, a native of Ireland, aged 25 years, unmarried, was admitted into the New York Hospital on the 6th Nov. 1848, at which time she had suffered from rheumatism already about seven months; all the larger joints of the body having been successively affected. About four months prior to her admission, she suffered a relapse, after having nearly recovered, and since then she has experienced but little alleviation of her ailments. Her neck as well as most of her larger joints are painful, though not much swollen. Her tongue is slightly furred, her pulse is 85 and soft; her skin moist and bowels regular.

On the 19th of January following, she had nearly recovered from her rheumatism, under treatment, except rigidity and contraction of the muscles of the right side of the neck, by which the head was drawn downwards and towards the right shoulder. To relieve this distortion, frictions with stimulating and oleaginous liniments were diligently employed, and, subsequently, sulphuric ether was applied to the neck. Some slight improvement resulted from the use of these means. On the 18th of April, however, the condition f the neck had for some time been stationary, and all hope of further benefit was abandoned. The motions of the head were very much restricted, and any attempt to overcome the resistance of the rigid muscles by stretching them occasioned severe pain. The rigidity did not appear to reside in the sterno-mastoid muscle, inasmuch as this muscle did not grow hard and stiff when efforts were made to elevate the head; the resistance was evidently seated in the deeper muscular and tendinous parts.

At the request of my colleague, Dr. Swett, of the Medical Division, I saw this patient and proposed to make cautious attempts to overcome the resistance by force, the patient being first subjected to the influence of sulphuric ether. Considering the resistance to depend on contracted muscular and tendinous fibres, my object was either to stretch or rupture them, and in doing this, no danger was apprehended to the important nerves and blood-vessels of the neck; since the forced movement necessary to accomplish this object would fall far short of the extensive motions in every direction to which these parts are accustomed naturally to accommodate themselves.

Doctor Swett assenting to my proposal, the patient was laid upon her back in bed, with her head resting high up on a pillow, so as to be easily got at from the head of the bed. Taking the head between my two hands placed one on either side, I cautiously stretched it with a very moderate degree of force in the direction opposite to that in which it was distorted, that is, upwards and to the left side. Almost immediately every one standing round the bed (of whom there were at least eight or ten pupils and medical men), was startled by a loud snapping sound of something rupturing, and at the same time I perceived that the head yielded, and could be brought almost to its natural position. It was thought prudent to proceed no further at this operation. The patient on recovering her consciousness was not sensible of any soreness in the parts, and could bear the head to be moved much easier than before the operation. She was directed to lie as much as possible on her left side. On the following day there was considerable soreness on the right side of the neck. On the 25th of April, one week after the first operation, the soreness of the neck having very much diminished, the operation was repeated a second time.

The proceedings were the same as in the first operation, only the stretching was carried to a much greater extent, and with a much less timid hand. Several times resisting fibres were felt to yield with a rupturing sensation, till, at length, no further resistance was encountered, and the head could be carried to the full extent in every direction. After the effects of the ether had passed off, the head was bandaged down towards the left shoulder. On the 1st of May, the bandage being dispensed with, the head showed no disposition to resume its distorted attitude. On the 10th of May (1849), the head could maintain, unaided, its erect natural position, though rotation and flexion were still limited in extent, and performed awkwardly; the patient however was sensible of the progressive improvement in these respects. She took her discharge from the Hospital for the purpose of returning to her friends in Ireland. About one year afterwards she was heard from as continuing well and free from any distortion or rigidity of the neck.

CASE SECOND.

In January, 1852, Maria P——, of Guilford, Connecticut, aged 12 years, and of a healthy constitution, came under my care, with the head very much distorted from being drawn down towards the chest, with the face turned to the left side. The motions of the head were also very much restricted. In the month of July preceding, she had been attacked with sore throat and stiff neck, that left her ever since in the condition just described. She had never suffered from rheumatism in any other part of her body, and had generally enjoyed good health. I at once decided to employ the treatment which had been so successful in the preceding case; and on the 15th of January, having first etherized my patient, I performed the first operation. In order to carry the extension of the head to the requisite degree, it became necessary to have her supported in the sitting posture in a chair, and to place myself in front of her. Grasping the head between my hands, I acted on it in the various directions in which resistance was encountered, but felt no sensation of rupturing fibres, in this or in any of the subsequent operations. The resisting parts, however, yielded in some measure, and allowed the head to be brought more nearly into its natural position. No pain was experienced from the operation on recovering her consciousness.

On the 19th, no effect was observable from the first operation; it was therefore repeated a second time, with the aid of ether. On the 24th, 26th and 30th of January, and on the 4th and 7th of February, it was also repeated, each time with the aid of ether. Though a gradual improvement was perceptible from these repeated operations, it became evident that a complete cure could only be achieved by a patient and persevering repetition of them for a long time; it was therefore judged most prudent to continue the operations without the aid of ether. The patient's courage and endurance, though put to a very severe test, proved adequate to the trial. Once every day she submitted with the most admirable fortitude to the stretching process, for about ten minutes each time. This was continued up to the 1st of March, after which it was repeated twice every day. The manner of manipulating was as follows: The patient was seated in a chair, and her body steadied by an assistant standing behind her and holding her shoulders firmly with both hands. Placing myself in front of her, I grasped her head with my hands in such a way as to perform most efficiently the different movements I wished to execute. These movements were varied in every direction in which resistance was encountered, my object being to stretch to the utmost the contracted muscles, and to maintain them on a stretch for a certain length of time. The process was painful only during its actual performance, and ceased to be so the moment it was discontinued. On the 24th of March, the operations were suspended, while the patient made a visit to her family, and were resumed again on the 8th of April. During this interval no relapse took place. The same course of treatment was continued till the 10th of May, when she returned to her home, highly gratified at being able to maintain her head by her own efforts in its natural erect position, and to turn it in different directions almost as well as ever she could. She was advised to continue for a long time the daily practice of performing the various motions of the head as extensively as possible. On the 13th of January, 1853, I conversed with an aunt of my patient, who had recently visited her, and who reports that she holds her head in a very natural manner, and can move it at pleasure freely in every direction. In a word, she considers herself quite well again, and without any disposition to relapse.

GURDON BUCK, M. D.,
Surgeon to New York Hospital.

121 Tenth-st. January 20, 1853.

